# Novel Chitosan-Based Schiff Base Compounds: Chemical Characterization and Antimicrobial Activity

**DOI:** 10.3390/molecules27092740

**Published:** 2022-04-24

**Authors:** Riccardo Fontana, Peggy Carla Raffaella Marconi, Antonella Caputo, Vasak B. Gavalyan

**Affiliations:** 1 Department of Chemical, Pharmaceutical and Agricultural Sciences, University of Ferrara, Via Fossato di Mortara 64b, 44121 Ferrara, Italy; fntrcr1@unife.it (R.F.); mcy@unife.it (P.C.R.M.); 2 A.I. Alikhanyan National Science Laboratory, Yerevan Physics Institute, 2-Alikhanyan Brothers St., Yerevan 0036, Armenia

**Keywords:** chitosan, chitosan-based Schiff base, antibacterial activity, antifungal activity

## Abstract

Chitosan (CS) and its derivatives are receiving considerable attention for their great biocompatibility and broad-spectrum activities in many fields. In this work, we aimed to characterize the antimicrobial activity of novel chitosan Schiff bases (CSSB). CS was synthesized by double deacetylation of chitin (Cn) after its extraction from the armors of crustaceans *Astacus leptodactylus*, and CSSB-1 and CSSB-2 were synthesized by interaction of CS with 4-(2-chloroethyl) benzaldehyde (aldehyde-1) and 4-(bromoethyl) benzaldehyde (aldehyde-2), respectively, at room temperature. The synthesized compounds were characterized by elemental analysis, gel permeation chromatography (GPC), infrared spectroscopy (FTIR), thermogravimetry (TG), and differential scanning calorimetry (DSC). The antimicrobial activity against Gram-positive (*Staphylococcus aureus*) and Gram-negative (*Pseudomonas aeruginosa*) bacteria and against yeasts (*Candida albicans*) was significantly increased due to their higher solubility as compared to unmodified CS opening perspectives for the use of these compounds for antimicrobial prevention in different fields as, for example, food industry, cosmetics, or restoration.

## 1. Introduction

The importance of finding new antimicrobial platforms stems from the study of antimicrobial resistance and the fact that microorganisms are widespread and can be responsible for major infections. Antibiotic resistance is currently the main threat to infectious diseases due to the epidemiological and economic impact of this phenomenon [1]. In an attempt to find therapeutic alternatives to combat microorganisms, no alternative is precluded: from synthetic molecules to plant extracts, from food processing of by-products to biofermentative processes, and even to urban waste. All these are potentially rich sources of bioactive molecules, such as antimicrobials, or molecules capable of altering the mechanisms of biofilm formation or capable of dissolving the matrix; in this perspective, chitosan (CS) and natural antimicrobial compounds could play a key role, so much so that CS is currently at the center of great international research interest. The use of natural compounds and medicinal plants dates back to ancient times, and chitosan, thanks to its multiple properties in different fields, could be a stimulating starting point for the discovery of new pharmacological/antimicrobial platforms. Chitosan has been studied as an antimicrobial material against a wide range of target organisms such as algae, bacteria, yeasts, and fungi in experiments involving in vivo and in vitro interactions with chitosan in different forms (solutions, films, and composites) [2]. Since the broad-spectrum antibacterial activity of this material was first proposed by Allan and Hardwiger [3], together with the great commercial potential, the antimicrobial property of chitosan and its derivatives has attracted great attention from researchers. With this in mind, we investigated the effect of CS and CSSB since the CSSBs reported so far exhibit better antimicrobial properties than bare chitosan.

Chitosan (CS) (β-(1 → 4)-2-amino-2-deoxy-d-glucopyranose) and its various derivatives are used in many fields due to their unique characteristics [4,5]. CS is prepared by deacetylation of chitin (β-(1 → 4)-2-acetamido-2-deoxy-d-glucopyranose), which is one of the most common natural polysaccharide biopolymers, inferior in nature only to cellulose. Among derivatives of CS, in our opinion, of particular interest are Schiff bases of chitosan (CSSB). Their synthesis is carried out via the reaction of the amino groups of CS with aldehydes or ketones. CS and some CSSB derivatives are inexpensive, non-toxic, highly biodegradable, biocompatible, and biologically active substances and are being used wildly in biomedicine, cosmetics, restoration, packaging of food, and environmental remediation. The biological activities of CSSB, including the antimicrobial properties, are important characteristics in terms of their specific use. Key physicochemical characteristics, such as average molecular weight (Mw), degree of N-acetylation (DA), polydispersity (Mw/Mn), crystallinity, and pattern of acetylation, are important determinants of CS and CSSB activities since they influence solubility in water and organic solvents [6,7,8,9,10].

The biological characteristics of CSSB synthesized on the basis of some aromatic aldehydes were recently investigated by several groups for their antimicrobial activity. CSSB was synthetized by reaction of CS with salicylaldehyde and showed that both the nature of the metal (Zn (II), Pd (II), and Pt (II)) complexes with the Schiff bases and the molecular weight of the starting CS (223 and 64 kDa) have a significant effect on the cytotoxicity, and in general on the antimicrobial activity of the compounds [11,12]. Based on 2-hydroxy-3-metoxybenzaldehyde, CSSB and its complex compounds with metal ions Cu (II), Ni (II), and Zn (II) were synthetized and shown to have better anticancer activity in vitro than pure CS [13]. In addition, acetophenone derivatives (4-aminoacetophenone enone and 4-bromoacetophenone) were used to synthesize CSSBs, and the antimicrobial activity of these chitosan derivatives was higher than unmodified CS, while their anticancer activity was inferior to CS [14]. In this paper, we present the results of our studies of CSSBs synthesized using aromatic aldehydes, namely 4-(2-chloroethyl) benzaldehyde (aldehyde-1) and 4-(2-bromoethyl) benzaldehyde (aldehyde-2) according to a previously described procedure [15]. In the reaction products (CSSB-1 and CSSB-2), there is an XCH_2_CH_2_ group capable of HX elimination reactions (HX = HCl, HBr) and the formation of pending vinyl groups [15]. By the reacting pending vinyl groups of CSSBs, novel polymer and copolymer systems with substantially valuable properties can be synthesized. Vinyl groups of CSSBs can also be considered as new potential centers for complexation (π complexes) with metal ions.

The starting CS was a double de-acetylated product with a degree of deacetylation of 81.7% and Mw of 84.5 kDa. By reacting CS with aldehyde-1 and aldehyde-2, CSSB-1 and CSSB-2 were synthesized, respectively, and physicochemically characterized by means of TG, FTIR, and GPC, as well as characterized for their antibacterial and antifungal activity.

## 2. Results and Discussion

### 2.1. Characteristics of Chitosan-Based Schiff Base Compounds

The CS used for the synthesis of CSSB-1 and CSSB-2 had a degree of deacetylation (DDA) of 81.7% and an average molecular weight value of Mn-48.8 kDa, Mw-84.5 kDa, and coefficient of polydispersity Mw/Mn 1.73. We believe that such a relatively low value of average molecular weight (AMW) was achieved by double deacetylation of the CS product. The scheme (Supplementary Materials, Figure S1) describes the synthesis of CSSB-1 and CSSB-2 by means of reaction of CS with aldehydes-1 and -2 (condensation reaction) to obtain the final products CSSB-1 and CSSB-2, respectively. Rather soft conditions of synthesis of CSSB-1 and CSSB-2 (temperature of the reaction environment or of condensation reaction 22 °C) suggest that values of AMW-s of CSSB-1 and CSSB-2 products do not undergo serious changes staying within limits Mn-48.8 kDa, Mw-84.5 kDa. This is important since the DDA and AMW values of CS and chitosan derivatives have a significant effect on many properties, including their biological activity.

The results of elemental analysis of the CS, CSSB-1, and CSSB-2 samples are shown in Table 1. The data from the elemental analysis show that in the tested samples, the percentages of carbon and nitrogen change noticeably. This is evident when comparing CS data with CSSB-1 and CSSB-2. Note that the fully de-acetylated CS contains 44.66% and 8.68% of carbon and nitrogen, respectively. The degree of substitution (DS) is an important characteristic for CS derivatives, including CSSB. The degree of substitution of hydrogen of the CS amino groups by the corresponding radical of aldehyde-1 or -2 is shown in the reaction of the scheme. The DS calculated by equation previously described [16] for CSSB-1 and CSSB-2 are 14.2% and 15.7%, respectively.

**Figure 1:**
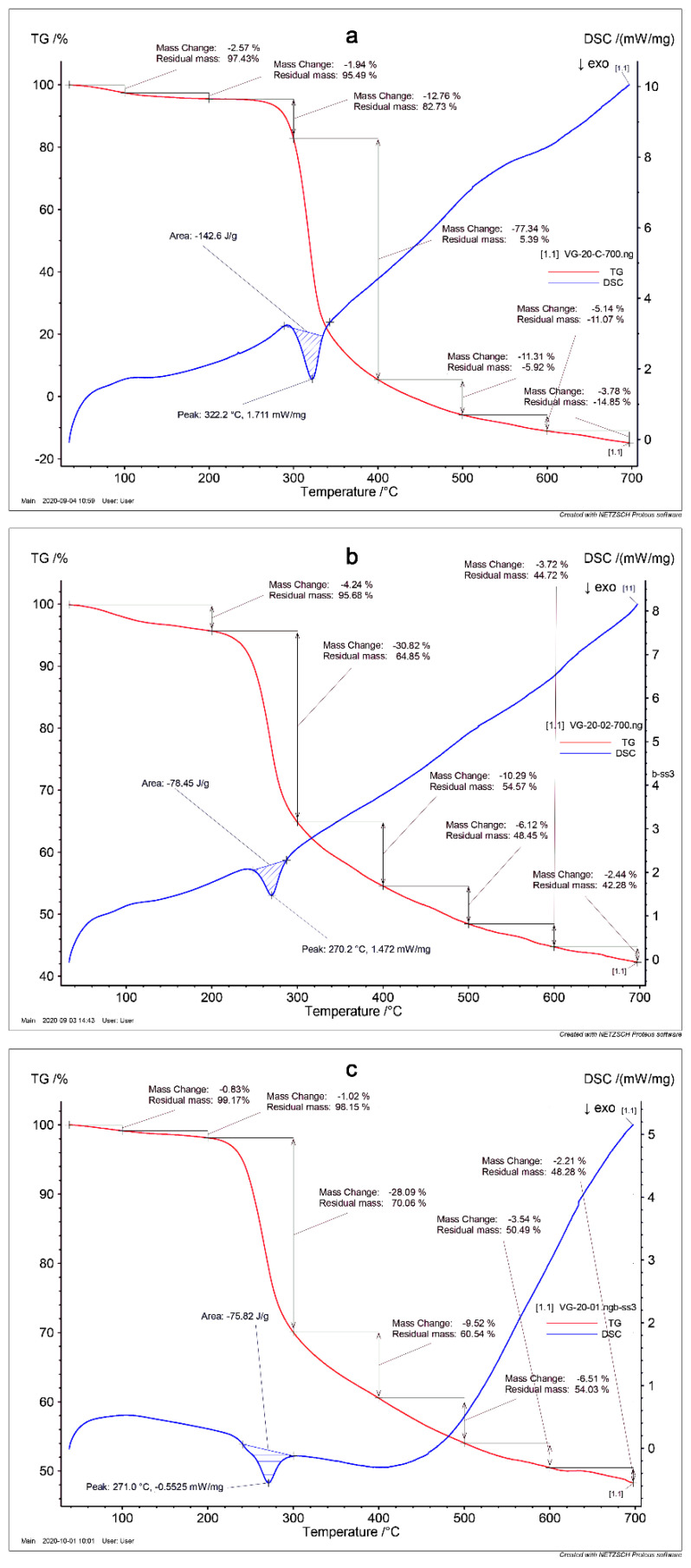
Characterization of the compounds. TG and DSC data of CS (**a**), CSSB−1 (**b**), and CSSB−2 (**c**) samples.

The data of thermogravimetry (TG) and differential scanning calorimetry (DSC) of CS, CSSB-1, and CSSB-2 samples are shown in Figure 1. It is noteworthy that intense weight loss (TG curves) for samples CSSB-1 and CSSB-2 (Figure 1b,c) starts at a lower temperature (200 °C) in comparison with sample CS (240 °C) (Figure 1a), while for all samples, CS, CSSB-1, and CSSB-2, it ends at temperatures of about 420 °C. The process of intense weight loss is accompanied by a single exothermic effect that clearly manifests itself on DSC curves. The exothermic effect for the CS sample (Figure 1a) begins at 287 °C and reaches the maximum temperature value (the minimum point on the DSC curve) at 322 °C. The starting temperatures and the minimum points on the DSC curve for samples CSSB-1 and CSSB-2 have values of 243, 270 °C and 239, 271 °C, respectively. Our results suggest that the thermal stability of CSSB relative to CS is significantly lower in agreement with other results [11,13,17]. We believe that in the synthesis of CS derivatives, the supramolecular structure of CS is destroyed, mainly due to numerous hydrogen bonds between macromolecules. The mobility of CS derivative macromolecules increases; thus, significantly less thermal energy is required to break down the weakest bonds compared to CS. In our opinion, it is this that can explain the fact that in terms of thermal stability, CSSB-1 and CSSB-2 are inferior to CS.

The main results of absorption spectra of the CS, CSSB-1, and CSSB-2 samples are summarized in Table 2 and Supplementary Figure S2. Attention is drawn to the lack of absorption in the region around 1651 cm^−1^ for samples of CSSB-1 and CSSB-2. We suggest that the absorption of the imine bond (C=N stretch, imine), which takes place with sufficient intensity in the region around 1640 cm^−1^, is inferior to the absorbance characteristic of the OH and NH2 (OH; NH2-stretch) and C-O-C and C-N (asymmetric stretch C-O-C and C-N stretch) groups. In fact, there is complete overlap in the absorption of the C=O bond (C=O stretch amide 1) in the region of about 1651 cm^−1^. As a result, in the ranges of absorption of samples CSSB-1 and CSSB-2, there is no absorption in the region near 1651 cm^−1^. The CS, CSSB-1, and CSSB-2 absorption spectra are in agreement with the data discussed by other groups [11,18,19,20,21].

### 2.2. Antimicrobial Properties of CS, CSSB-1, and CSSB-2

In order to assess and compare the antimicrobial activity of CS and its Schiff bases derivatives, CSSB-1 and CSSB-2, two bacterial strains (i.e., the Gram-negative *P. aeruginosa* and the Gram-positive *S. aureus*) and one yeast strain (*C. albicans*) were used as model microorganisms. They were incubated with three different concentrations (0.006%, 0.012%, and 0.024%) of each compound for 6, 18, and 24 h to determine both the lower effective dose and a time-dependent effect. The antimicrobial activity of CS, CSSB-1, and CSSB-2 was measured as the reduction in growth (i.e., reduction in colony-forming units/mL (CFU/mL) and the reduction in the optical density at 600 nm compared to each untreated microorganism).

**Figure 2:**
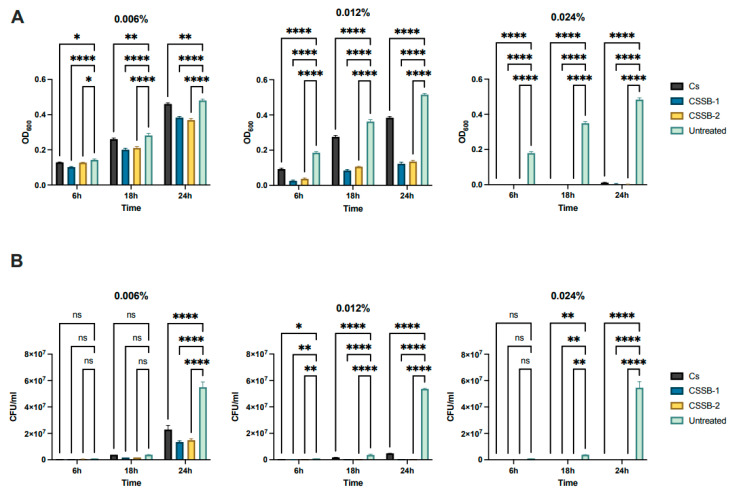
Antimicrobial activity of CS, CSSB-1, and CSSB-2 compounds on *S. Aureus*. Fresh cultures of *S. aureus* were incubated in liquid cultures with three different concentrations (0.006%, 0.012%, and 0.024%) of CS, CSSB-1, and CSSB-2 for 6, 18, and 24 h or without the compounds (untreated cells). After incubation, the antimicrobial activity was evaluated by measuring the optical density at 600 nm of each liquid culture (**A**) and by determining the number of colony-forming units/mL (CFU/mL) (**B**) after plating the cultures onto agar plates and incubation to allow colony formation. *, *p* = 0.01; **, *p* = 0.001; ****, *p* < 0.0001.

**Figure 3:**
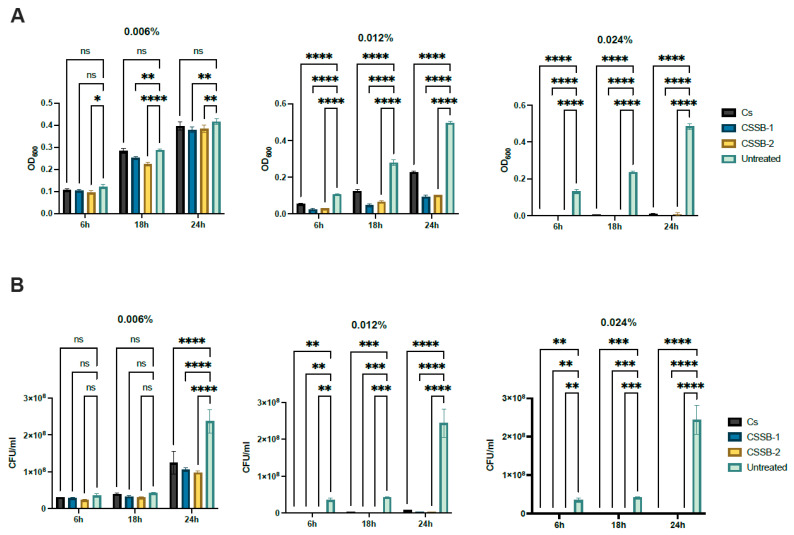
Antimicrobial activity of CS, CSSB-1, and CSSB-2 compounds on *P. aeurginosa*. Fresh cultures of *P. aeruginosa* were incubated with three different concentrations (0.006%, 0.012%, and 0.024%) of CS, CSSB-1, and CSSB-2 for 6, 18, and 24 h or without the compounds (untreated cells). After incubation, the antimicrobial activity was evaluated by measuring the optical density at 600 nm of each liquid culture (**A**) and by determining the number of colony-forming units/mL (CFU/mL) (**B**) after plating the cultures onto agar plates and incubation to allow colony formation. *, *p* = 0.01; **, *p* = 0.001; ***, *p* = 0.0001; ****, *p* < 0.0001.

**Figure 4:**
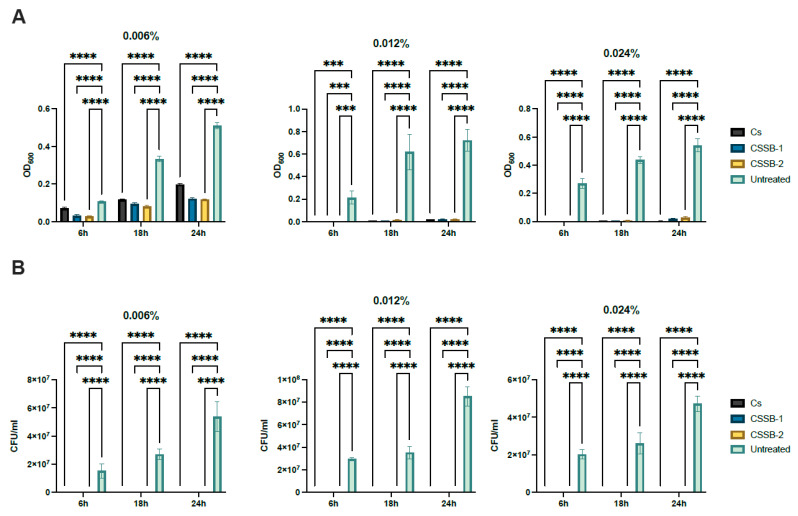
Antimicrobial activity of CS, CSSB-1, and CSSB-2 compounds on *C. albicans*. Fresh cultures of *C. albicans* were incubated with three different concentrations (0.006%, 0.012%, and 0.024%) of CS, CSSB-1, and CSSB-2 for 6, 18, and 24 h or without the compounds (untreated cells). After incubation, the antimicrobial activity was evaluated by measuring the optical density at 600 nm of each liquid culture (**A**) and by determining the number of colony-forming units/mL (CFU/mL) (**B**) after plating the cultures onto agar plates and incubation to allow colony formation. ***, *p* = 0.0001; ****, *p* < 0.0001.

**Figure 5:**
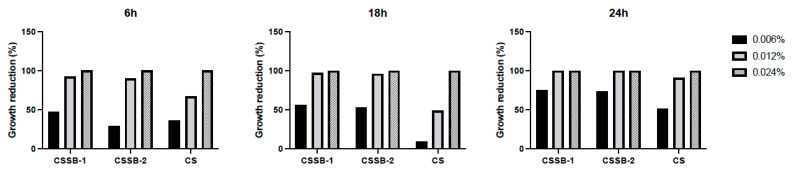
Growth reduction in *S. Aureus* after treatment with CS, CSSB-1, and CSSB-2 compounds. Fresh cultures of *S. aureus* were incubated in liquid cultures with three different concentrations (0.006%, 0.012%, and 0.024%) of CS, CSSB-1, and CSSB-2 for 6, 18, and 24 h or without the compounds (untreated cells). After incubation, each culture was plated onto agar plates and incubated to allow colony formation (CFU/mL, as illustrated in Figure 2). The results are represented as percentage (%) of cell growth reduction after each treatment as compared to control, untreated cells.

**Figure 6:**
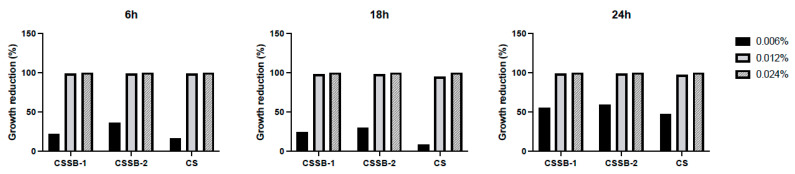
Growth reduction in *P. aeruginosa* after treatment with CS, CSSB-1, and CSSB-2 compounds. Fresh cultures of *P. aeruginosa* were incubated in liquid cultures with three different concentrations (0.006%, 0.012%, and 0.024%) of CS, CSSB-1, and CSSB-2 for 6, 18, and 24 h or without the compounds (untreated cells). After incubation, each culture was plated onto agar plates and incubated to allow colony formation (CFU/mL, as illustrated in Figure 3). The results are represented as percentage (%) of cell growth reduction after each treatment as compared to control, untreated cells.

**Figure 7:**
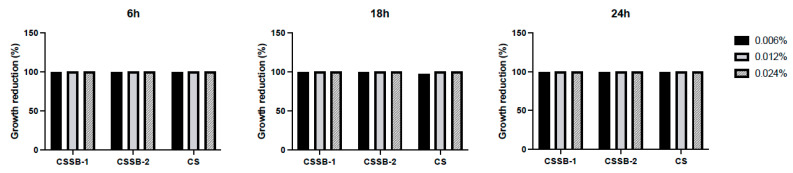
Growth reduction in *C. albicans* after treatment with CS, CSSB-1, and CSSB-2 compounds. Fresh cultures of *C. albicans* were incubated in liquid cultures with three different concentrations (0.006%, 0.012%, and 0.024%) of CS, CSSB-1, and CSSB-2 for 6, 18, and 24 h or without the compounds (untreated cells). After incubation, each culture was plated onto agar plates and incubated to allow colony formation (CFU/mL, as illustrated in Figure 4). The results are represented as percentage (%) of cell growth reduction after each treatment as compared to control, untreated cells.

As shown in Figure 2, Figure 3 and Figure 4 and Supplementary Tables S1–S3, as observed by the numbers of alive cells (CFU/mL) and the absorbance values of the cultures, in general, the antimicrobial activity of the CS, CSSB-1, and CSSB-2 compounds was inversely related to their concentration at each time point and for each microorganism. Of note, for *S. aureus,* the effect was also inversely related to the time of contact since at the lower dose of 0.006%, the reduction in bacterial growth was higher after 24 h of contact (for all compounds, 100% of growth reduction compared to untreated cells) than at shorter time points of 6 h (CS: 36%; CSSB-1: 47%; CSSB-2: 29%) and 18 h (CS: 67%; CSSB-1: 92%; CSSB-2: 90%) (Figure 5, Figure S3 and Table S4). A slightly different result was observed for *P. aeruginosa* as, in general, the lower dose was less effective. In fact, although the antibacterial effect of the CS, CSSB-1, and CSSB-2 compounds was inversely related to their concentration at each time point, as for *S. aureus*, however, all three compounds at 0.006% were less effective as compared to *S. aureus*, in controlling the growth of *P. aeruginosa* at all-time points, whereas at the higher concentration (0.024%) 100% inhibition of *P. aeruginosa* growth was observed in a fashion similar to *S. aureus* (Figure 6, Figure S4 and Table S5). These results suggest a different behavior of CS and CSSBs depending on the nature and structure of the outer surface of the Gram-positive and Gram-negative bacterial cells. The lower antibacterial activity at dose of 0.006% observed on *P. aeruginosa* suggest that the outer membrane of Gram-negative cells creates a barrier, likely reducing the entry of the molecules and thus their effect on cell growth. That the chemical structure of the cell surface may have a role in modulating the antimicrobial activity of CS, CSSB-1, and CSSB-2 is also suggested by the observation that all three compounds showed significantly greater inhibitory activity on yeast than bacterial cells. In fact, almost 100% reduction in *C. albicans* growth was observed even at the lowest concentration of 0.006% already after 6 h of contact. This may depend on the different chemical compositions of the walls of yeasts and bacteria, made of chitin and peptidoglycan, respectively (Figure 7, Figure S5, and Table S6). It is plausible to hypothesize that these compounds (CS, CSSB-1, and CSSB-2) compete with chitin precursors and interfere with the synthesis of the yeast wall, inhibiting the growth of yeast cells very efficiently.

Finally, our results indicate that the CS Schiff base derivatives, CSSB-1 and CSSB-2, exerted higher antimicrobial activity than unmodified CS as compared to the untreated cultures. The action of these compounds may be related to their positive charged amino groups binding to the surface of bacterial wall or membrane, which are negatively charged, leading to alteration of cell permeability and cell death. This is particularly evident against *S. aureus* and *P. aeruginosa* at all time points with doses of 0.006% and 0.012%. The greater activity might be due to the higher water solubility of CSSB-1 and CSSB-2 compounds, allowing for their almost complete suspension, and to the substitutions themselves (Figure 5, Figure 6 and Figure 7, Figures S3–S5, and Table S4–S6).

Other studies showed that modification of CS can increase its antimicrobial activity. CS modified with acetophenone derivatives through Schiff base reaction possesses increased efficacy due to its electron-donating nature [14]. Recently, it was also reported that a new CSSB (3EtO-4OH/Chit) and its 3EtO-4OH/Chit/Fe_2_O_3_ nanocomposite have greater antimicrobial activity than CS itself with 3EtO-4OH/Chit/Fe_2_O_3_ showing the highest activity due to the presence of Fe_2_O_3_ nanoparticles, which increase the total antimicrobial property of 3EtO-4OH/Chit [20]. Yin et al. tried an approach similar to ours [22] and showed that CSSBs synthetized from an aldehyde showed better antimicrobial properties, thanks to these electron-withdrawing substituents. Our findings and data from the literature allow us to state that these substitutions enhance the antibacterial activity of CS-based products.

We are proceeding with work on the synthesis and characterization of completely new O-carboxymethyl derivatives of CSSB-1 and CSSB-2 and with the study of their antimicrobial properties.

## 3. Materials and Methods

### 3.1. Reagents, Instruments, and Products

The materials and methods used are described by Gavalyan [15]. The molecular weights of the CS samples were obtained by Gel Penetration Chromatograph (GPC) Agilent 1200 (Agilent Technologies, Santa Clara, CA, USA) columns U-Hydrogel 500 and 250 (Waters), T = 25 °C. Elemental analyses (C, H, N) were performed on Euro EA 3000 analyzer (Euro Vector, Pavia, Italy). Attenuated total reflection Fourier-transform infrared spectra (FTIR ATR) was registered by an FTIR Microscope LUMOS (Bruker, Billerica, MA, USA) spectrometer (Ge prism, Happ-Genzel apodization, ATR distortion is corrected, number of scans 32; resolution: 4 cm^−1^). The thermogravimetry (TG) was carried out on STA 449 F3 JUPITER from Netzsch (Selb, Germany), helium atmosphere (the flow rate is 20 mL/s), temperature gradient from room temperature to 700 °C/10 °C/min, the initial weight of each sample was 10 mg.

The extraction of Cn from armors of crayfish *Astacus leptodactylus* (Lake Sevan, Armenia), the synthesis of CS, aromatic aldehydes 4-(2-chloroethyl) benzaldehyde (aldehyde-1), 4-(2-bromoethyl) benzaldehyde (aldehyde-2) and chitosan Schiff bases (CSSB-1 and CSSB-2) were carried out as described [15]. The elemental analysis of CS after the second round of deacetylation was C, 45.37%, N, 8.31%, and H, 6.73%. The degree of acetylation (DA) of synthesized CS (18.3%) was calculated by using elemental analysis data and an equation reported by Kasaai et al. [23]. The yields of CSSB-1 and CSSB-2 synthesized at 22 °C (RT) were 17.3% and 19.4%, respectively. CSSB-1 and CSSB-2 were lyophilized and sent to the University of Ferrara for antimicrobial activity testing. For this purpose, they were dissolved in sterile water at 10 mg/mL for 24 h at 37 °C, filtered through 0.22 µm filters (Starlab, Milan, Italy), aliquoted, and stored at RT until use.

### 3.2. Bacterial Strains and Culture Conditions

Bacteria and yeast isolates were purchased from ATCC (*S. aureus* (ATCC 25923), *P. aeruginosa* (ATCC 89033) and *C. albicans* (ATCC 90028)) and stored at −80 °C in Luria-Bertani (LB) broth (Liofilchem, Roseto degli Abruzzi, TE, Italy) with 50% glycerol. The experiments were carried out with bacteria cultured in Tryptic Soy Broth (TSB) whereas yeasts in Sabouraud Dextrose Broth (SDB) (Liofilchem) in agitation at 150 rpm (MaxQ 4000, Thermo Scientific, Milan, Italy) or plated on Tryptic Soy Agar (TSA) (Scharlab Italia, Milan, Italy) or Sabouraud Dextrose Agar (SDA) (Liofilchem), respectively, and incubation at 35 °C/37 °C (bacteria) and 25 °C (yeasts).

### 3.3. Determination of the Antimicrobial Activity of CSSB

To determine the antimicrobial activity of the CSSB products, microorganisms were cultured in 5 mL of TSB or SDB broth for 18 h and, after incubation, the absorbance values of the cultures were read at 600 nm (OD_600_) with a spectrophotometer to calculate the number of cells/mL using the formula “sample OD_600_ × 10^6^/0,1”. For *P. aeruginosa* and *S. aureus* cultures, the values were further multiplied by 70 and 100, respectively, to obtain the real concentration of cells/mL. The optical density, in fact, underestimates the number of *P. aeruginosa* and *S. aureus* bacterial cells in liquid media. Thus, each bacterial strain was titrated on agar plates, and in parallel, the bacterial inocula were read with the spectrophotometer to determine the conversion factors (70 and 100, respectively, for *P. aeruginosa* and *S. aureus*). Microorganisms (5 × 10^2^ cells/mL) were then diluted 10^−3^ and 10^−4^ in a liquid medium and incubated (3 mL of each dilution in 10 mL tubes) with CS, CSSB-1, and CSSB-2 at three different final concentrations (0.006%, 0.012%, and 0.024%) or without any compound (untreated control) for three different time points (6, 18 and 24 h). Following incubation, the tubes were observed for the presence of turbidity and the absorbance (OD_600_) of each tube measured. Each sample (1 mL) was then plated in duplicate on the surface of TSA or SDA plates (undiluted or previously diluted 10^−1^ up to 10^−8^) and incubated for 24 h (bacteria) and 2–5 days (yeasts). Where present, colonies were counted and reported as colony-forming units (CFU) per mL. For each compound concentration and for each time point, the analyses were performed on the results of three independent experiments and are represented as the mean ± SD.

### 3.4. Statistical Analysis

Statistical analysis was performed using one-way ANOVA followed by Dunnett’s multiple comparisons test with GraphPad Prism version 9.0.0 for MacOS (GraphPad Software, San Diego, CA, USA), with *p* ≤ 0.05 to identify significant differences. *, *p* = 0.01; **, *p* = 0.001; ***, *p* = 0.0001; ****, *p* < 0.0001.

## 4. Conclusions

CS was synthesized from Cn, and then by its interaction with aromatic aldehydes, novel chitosan Schiff bases (CSSB) were produced and structurally characterized. We have shown that the thermal stability of CSSB is inferior, and the supramolecular structure of CS is destroyed, mainly due to numerous hydrogen bonds between macromolecules. The mobility of CS derivative macromolecules increases; thus, significantly less thermal energy is required to break down the weakest bonds compared to CS. The antibacterial and antifungal activity of the novel compounds (CSSB-1 and CSSB-2) have significantly improved due to their higher solubility as compared to CS.

## Figures and Tables

**Table 1:** Elemental analysis data of Cn, CS, CSSB-1, and CSSB-2.

Sample	Obtained				Calculated ^a^							Yield %
	%C	%N	%H	C/N	%C	%N	%H	%O	% Cl	% Br	C/N	
Cn, DA 90.5%	46.33	6.98	6.36	6.64	47.09	7.03	6.48	39.40	-	-	6.70	-
CS, DA 18.3%	45.37	8.31	6.73	5.46	45.28	8.29	6.80	39.63	-	-	5.46	-
CSSB-1	48.13	7.54	6.37	6.38	47.92	7.47	6.59	35.68	2.35	-	6.41	17.3
CSSB-2	47.00	6.86	6.25	6.85	46.80	7.05	6.32	33.68	-	6.16	6.77	19.4

^a^ In calculations, the content of water is not considered.

**Table 2:** FTIR data of CS, CSSB-1, and CSSB-2.

Vibration Type in Group and Its Wavenumber, cm^−1^							
Sample	OH; NH_2_ Stretch	C=N Stretch (Imine)	C-H Stretch (CH_2_)	C=O Stretch Amide 1	C=C; C-H Stretch Aromatic Ring	Asymmetric Stretch C-O-C and C-N Stretch	O-bridge Stretch (Glucosamine)
CS	3362.5 3296.2	-	2874.5	1651.9	-	1150.7 1081.7	1028.7
CSSB-1	3367.7 3296.3	1639.4	2871.5	-	1602.1; 1522.1 1454.5	1150.4 1064.3	1030.1
CSSB-2	3364.6 3306.8	1641.0	2875.0	-	1609.2; 1518.2 1447.8	1150.3 1067.9	1025.5

## Data Availability

The data are presented in the text and in supplementary material.

## Supplementary Materials

The following supporting information can be downloaded at: https://www.mdpi.com/article/10.3390/molecules27092740/s1, Tables S1–S6 and Figures S1–S5.

## Sample Availability

Samples of the compounds are not available from the authors.
