# Supplementary material for: Novel Chitosan-Based Schiff Base Compounds: Chemical Characterization and Antimicrobial Activity
Source: Molecules. 2022 Apr 24;27(9):2740. doi: 10.3390/molecules27092740 (PMC9102824; doi:10.3390/molecules27092740)
Supplement: Supplementary file 1 [file molecules-27-02740-s001.zip › molecules-1693557-supplementary.pdf]

Supplementary

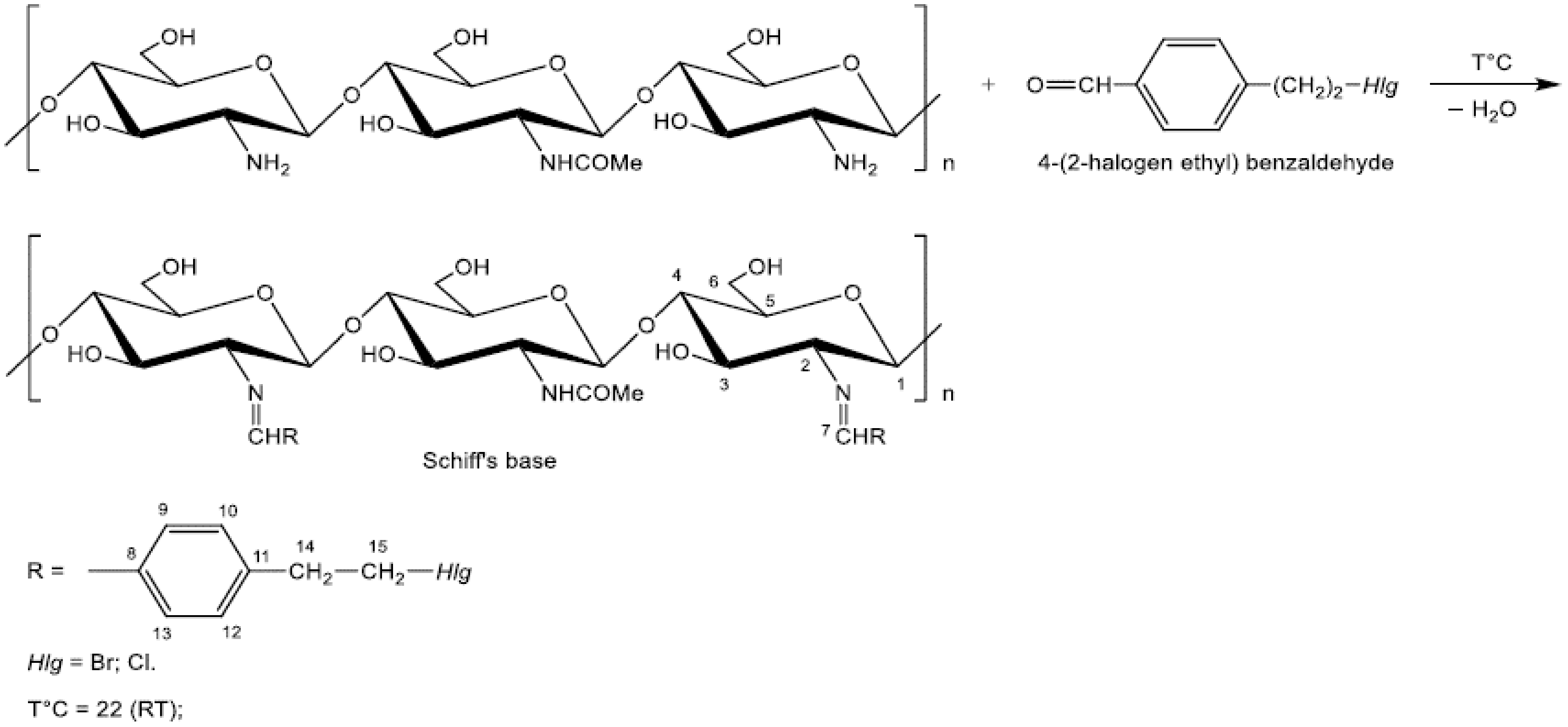

Figure S1. Scheme of the synthesis of CSSB by interaction of CS with 4-(2-halogenethyl)benzaldehyde.

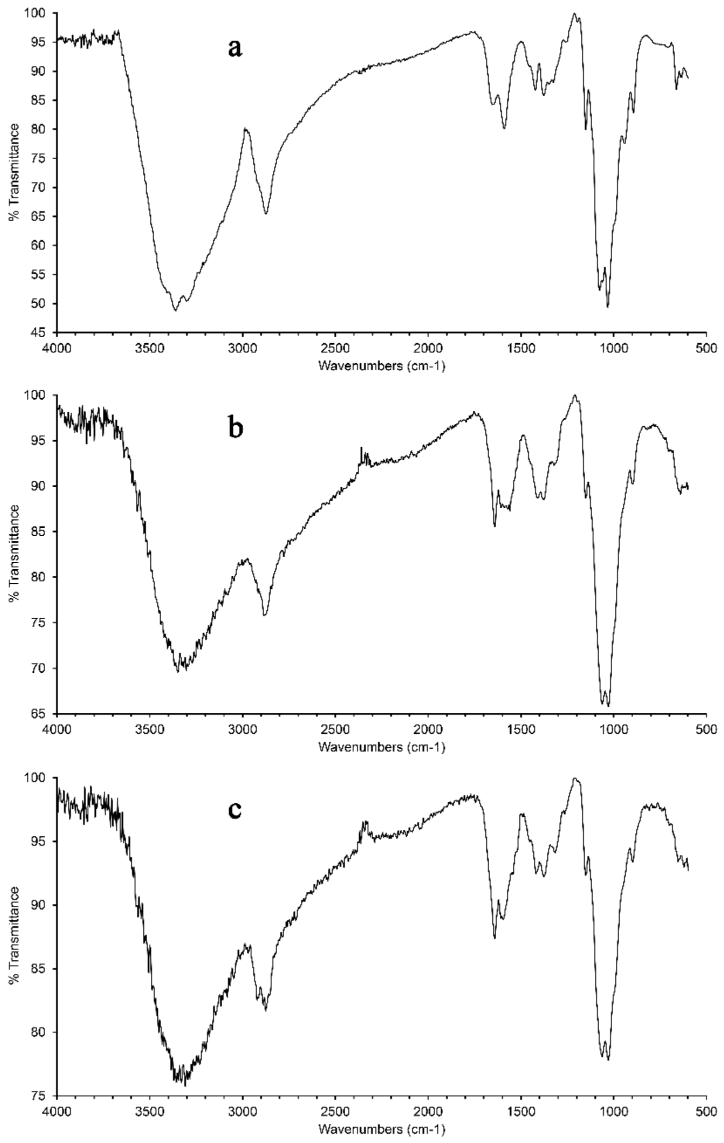

Figure S2. Characterization of the compounds. FTIR spectra of CS (a), CSSB-1 (b) and CSSB-2 (c) samples.

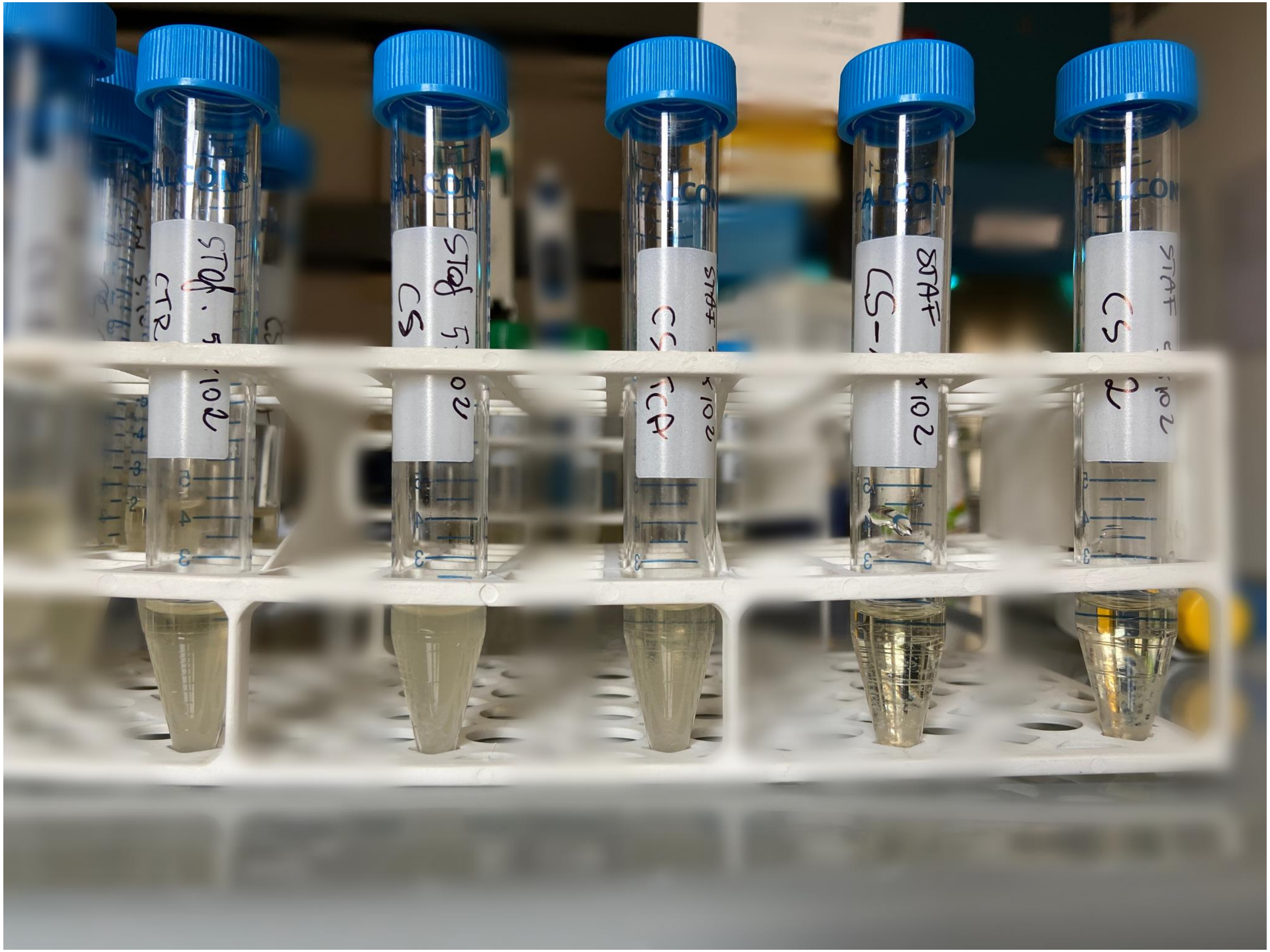
FALCON

Figure S3. Cultures of *S. aureus* with and without the compounds (CS, CSSB-1, CSSB-2). The picture of overnight cultures of *S. aureus*, untreated (CTR) and treated with CS, CSSB-1(CS-1) and CSSB-2 (CS-2) is shown. The sample labeled as CS-TCA refers to *S. aureus* treated with CS dissolved in trichloroacetic acid (TCA) instead of water. One representative experiment is shown.

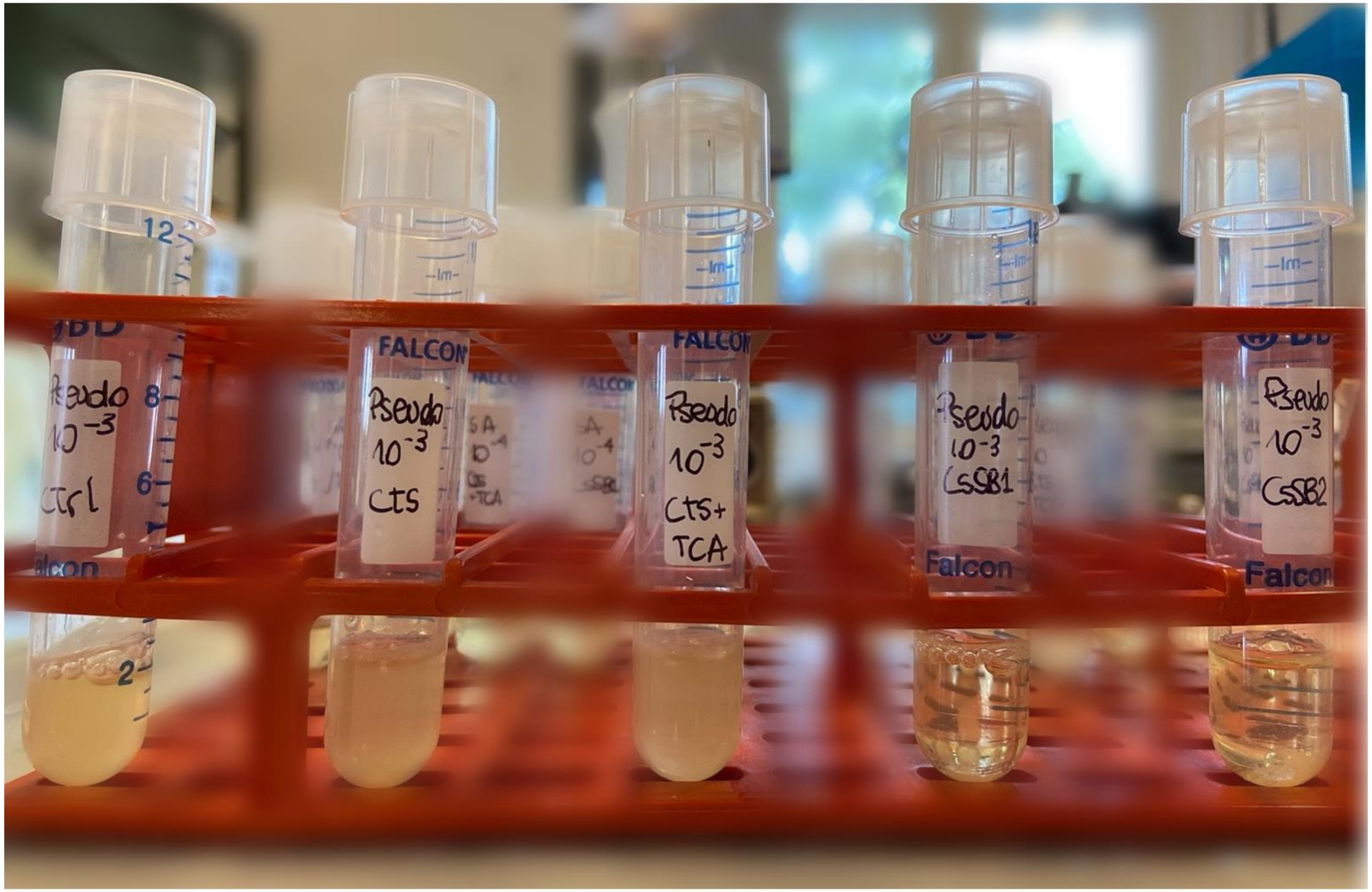

Figure S4. Cultures of *P. aeruginosa* with and without the compounds (CS, CSSB-1, CSSB-2). The picture of overnight cultures of *P. aeruginosa*, untreated (CTRL) and treated with CS (Cts), CSSB-1and CSSB-2 is shown. The sample labeled as Cts-TCA refers to *P. aeruginosa* treated with CS dissolved in TCA instead of water. One representative experiment is shown.

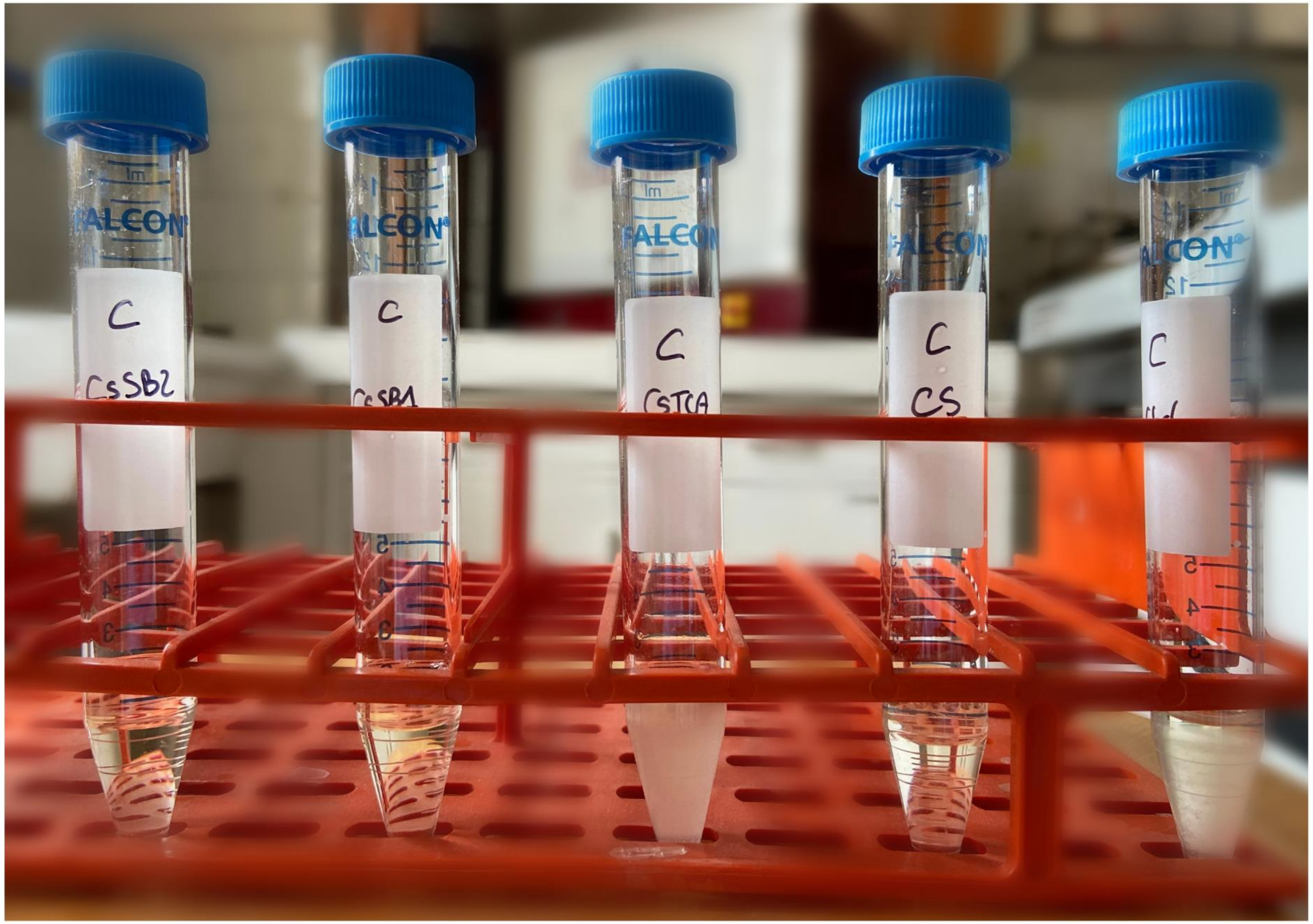

Figure S5. Cultures of *C. albicans* with and without the compounds (CS, CSSB-1, CSSB-2). The picture of overnight cultures of *C. albicans*, untreated (CTRL) and treated with CS, CSSB-1and CSSB-2 is shown. The sample labeled as CS-TCA refers to *C. albicans* treated with CS dissolved in TCA instead of water. One representative experiment is shown.

Table S1: Effect of CS and CSSBs at different concentrations after 6 hours

Sample	*S. aureus*						*P. aeruginosa*						*C. albicans*					
	**CFU/ml (mean ± SD)**			**OD_{600} (mean ± SD)**			**CFU/ml (mean ± SD)**			**OD_{600} (mean ± SD)**			**CFU/ml (mean ± SD)**			**OD_{600} (mean ± SD)**		
Concentration	0,006 %	0,012 %	0,024 %	0,006 %	0,012 %	0,024 %	0,006%	0,012 %	0,024%	0,006 %	0,012 %	0,024 %	0,006%	0,012%	0,024%	0,006 %	0,012 %	0,024 %
CSSB-1	403.666 ± 46.090	64.266 ± 4.148	0	0,102 ± 0,003	0,026 ± 0,004	0	27.700.000 ± 89.296	337.333 ± 8.020	0	0,103 ± 0,006	0,026 ± 0,006	0	36.333 ± 4.725	0,5 ± 0,5	0	0,031 ± 0,006	0	0
CSSB-2	542.000 ± 163.789	75.866 ± 1.703	0	0,126 ± 0,002	0,037 ± 0,004	0	22.666.666 ± 67.644	371.666 ± 820	0	0,094 ± 0,008	0,030 ± 0,002	0	20.833 ± 2.466	1 ± 1	0,666 ± 0,288	0,027 ± 0,003	0	0
CS	484.666 ± 142.945	248.333 ± 28.431	7 ± 4	0,128 ± 0,002	0,093 ± 0,005	0	30.036.666 ± 52915	416.666 ± 35.118	28 ± 3	0,106 ± 0,007	0,055 ± 0,005	0,002 ± 0,001	63.666 ± 5.131	0,166 ± 0,288	0,833 ± 0,288	0,07 ± 0,009	0	0
Untreated	761.666 ± 100.041	760.000 ± 9.165	762.666 ± 62.681	0,183 ± 0,005	0,186 ± 0,010	0,18 ± 0,008	35.620.000 ± 208.166	33.886.666 ± 130.912	34.886.666 ± 56.862	0,121 ± 0,010	0,108 ± 0,010	0,131 ± 0,013	15.333.333 ± 5.131.601	29.533.666 ± 6.771.509	20.233.333 ± 3.257.811	0,205 ± 0,005	0,213 ± 0,059	0,269 ± 0,034

Table S2: Effect of CS and CSSBs at different concentrations after 18 hours

Sample	*S. aureus*						*P. aeruginosa*						*C. albicans*					
	CFU/ml (mean ± SD)			OD_{600} (mean ± SD)			CFU/ml (mean ± SD)			OD_{600} (mean ± SD)			CFU/ml (mean ± SD)			OD_{600} (mean ± SD)		
Concentration	0,006%	0,012%	0,024%	0,006%	0,012%	0,024%	0,006%	0,012%	0,024%	0,006%	0,012%	0,024%	0,006%	0,012%	0,024%	0,006%	0,012%	0,024%
CSSB-1	1.686.666 ± 32.145	102.666 ± 2.516	0	0,201 ± 0,008	0,084 ± 0,006	0	32.100.000 ± 182.482	983.666 ± 7.505	0	0,251 ± 0,008	0,047 ± 0,010	0	93.333 ± 10.785	0,333 ± 0,577	0,833 ± 0,273	0,094 ± 0,006	0,009 ± 0,001	0,006 ± 0,001
CSSB-2	1.829.333 ± 25.704	123.883 ± 2.826	0	0,210 ± 0,009	0,096 ± 0,002	0	29.400.000 ± 228.703	721.333 ± 11.547	0	0,224 ± 0,008	0,066 ± 0,005	0	79.666 ± 1.016	1,5 ± 0,5	0	0,081 ± 0,005	0,015 ± 0,005	0,005 ± 0,004
CS	3.543.333 ± 30.550	1.750.333 ± 71.178	18 ± 9	0,261 ± 0,006	0,275 ± 0,008	0	39.000.000 ± 235.094	1.971.666 ± 57.518	74 ± 3,6	0,283 ± 0,011	0,125 ± 0,007	0,004 ± 0,001	103.000 ± 5.291	2 ± 1	9,8 ± 2,363	0,116 ± 0,005	0,005 ± 0,003	0,005 ± 0,001
Untreated	3.873.333 ± 93.408	3.440.666 ± 34.297	3.795.666 ± 85.165	0,282 ± 0,012	0,363 ± 0,007	0,349 ± 0,009	42.224.666 ± 209.505	42.546.666± 17.512	42.213.333 ± 165.702	0,288 ± 0,005	0,28 ± 0,014	0,236 ± 0,085	27.000.000 ± 3.605.551	35.333.333 ± 5.507.571	26.066.666 ± 5.704.676	0,433 ± 0,015	0,518 ± 0,157	0,436 ± 0,026

Table S3: Effect of CS and CSSBs at different concentrations after 24 hours

Sample	*S. aureus*						*P. aeruginosa*						*C. albicans*					
	CFU/ml (mean ± SD)			OD_{600} (mean ± SD)			CFU/ml (mean ± SD)			OD_{600} (mean ± SD)			CFU/ml (mean ± SD)			OD_{600} (mean ± SD)		
Concentration	0,006%	0,012%	0,024%	0,006%	0,012%	0,024%	0,006%	0,012%	0,024%	0,006%	0,012%	0,024%	0,006%	0,012%	0,024%	0,006%	0,012%	0,024%
CSSB-1	13.450.000 ± 921.832	128.000 ± 3.000	0	0,383 ± 0,007	0,102 ± 0,010	0,003 ± 0,001	106.800.000 ± 602.771	3.126.666 ± 110.151	0	0,379 ± 0,012	0,094 ± 0,008	0	118.000 ± 11.789	37,833 ± 33,43	0	0,122 ± 0,005	0,007 ± 0,010	0,018 ± 0,008
CSSB-2	14.803.333 ± 110.086	150.466 ± 11.772	0	0,369 ± 0,008	0,105 ± 0,007	0,002 ± 0,001	97.433.333 ± 896.288	3.367.000 ± 98.422	0	0,384 ± 0,018	0,102 ± 0,003	0,006 ± 0,001	112.333 ± 7.505	2 ± 1	3,666 ± 4,041	0,118 ± 0,002	0,021 ± 0,004	0,027 ± 0,012
CS	26.596.666 ± 195.533	4.736.666 ± 100.166	28 ± 36	0,461 ± 0,006	0,384 ± 0,007	0,011 ± 0,008	124.333.333 ± 280.555	7.013.333 ± 130.511	90 ± 5,9	0,397 ± 0,018	0,228 ± 0,006	0,010 ± 0,002	313.666 ± 13.576	0,666 ± 0,577	0,333 ± 0,263	0,197 ± 0,006	0,017 ± 0,003	0,003 ± 0,003
Untreated	54.700.000 ± 1.100.000	54.500.000 ± 1.212.436	54.333.333 ± 1.000.000	0,480 ± 0,008	0,515 ± 0,015	0,486 ± 0,010	236.666.666 ± 2.360.555	243.700.000 ± 1.400.000	235.000.000 ± 2.646.000	0,416 ± 0,013	0,485 ± 0,015	0,525 ± 0,058	53.666.666 ± 10.598.742	85.000.000 ± 8.660.254	47.100.000 ± 4.028.647	0,511 ± 0,018	0,622 ± 0,096	0,541 ± 0,046

Table S4. Percentage (%) reduction of CFU/ml versus untreated cells (*S. aureus*)

Sample	6h			18h			24h		
	**0.006%**	**0.012%**	**0.024%**	**0.006%**	**0.012%**	**0.024%**	**0.006%**	**0.012%**	**0.024%**
CSSB-1	47%	92%	100%	56%	97%	100%	75%	100%	100%
CSSB-2	29%	90%	100%	53%	96%	100%	73%	100%	100%
CS	36%	67%	100%	9%	49%	100%	51%	91%	100%

Table S5. Percentage (%) reduction of CFU/ml versus untreated cells (*P. aeruginosa*)

**Sample**	**6h**			**18h**			**24h**		
	**0.006%**	**0.012%**	**0.024%**	**0.006%**	**0.012%**	**0.024%**	**0.006%**	**0.012%**	**0.024%**
CSSB-1	22%	99%	100%	24%	98%	100%	55%	99%	100%
CSSB-2	36%	99%	100%	30%	98%	100%	59%	99%	100%
CS	16%	99%	100%	8%	95%	100%	47%	97%	100%

Table S6. Percentage (%) reduction of CFU/ml versus untreated cells (*C. albicans*)

Sample	6h			18h			24h		
	**0.006%**	**0.012%**	**0.024%**	**0.006%**	**0.012%**	**0.024%**	**0.006%**	**0.012%**	**0,024%**
CSSB-1	99%	100%	100%	99%	100%	100%	99%	100%	100%
CSSB-2	99%	100%	100%	99%	100%	100%	99%	100%	100%
CS	99%	100%	100%	97%	100%	100%	99%	100%	100%
